# Revisiting lymphatic dissemination in gastric cancer: insights from a large Western multicentre cohort

**DOI:** 10.1093/bjsopen/zrag094

**Published:** 2026-07-03

**Authors:** Marcus Fernando Kodama Pertille Ramos, Rubén Aguilar Zapag, Carlos García Carrasco

**Affiliations:** Department of Gastroenterology, University of São Paulo Medical School, São Paulo, Brazil; President of the Latin American Chapter of the International Gastric Cancer Association, Asunción, Paraguay; President of the 17th International Gastric Cancer Congress 2027, Santiago, Chile

The fundamental principle of gastric cancer surgery is achieving an oncologic resection with adequate margins combined with lymphadenectomy of nodal stations at risk for metastatic involvement. Over the years, the extent of lymphadenectomy has been largely defined by Japanese guidelines^1^, which established the nodal stations included in a D2 lymphadenectomy, currently considered the standard approach for the vast majority of patients. With the growing interest in more functional and less extensive procedures, such as proximal gastrectomy and pylorus-preserving gastrectomy, a detailed understanding of lymphatic dissemination patterns has become increasingly important. The publication by Kurokawa *et al*.^2^ evaluating lymph node involvement in oesophagogastric junction tumours further highlights the importance of this knowledge in defining the optimal surgical approach and extent of resection.

The recognition of the biological heterogeneity of gastric cancer across different populations worldwide raises an important question: does lymph node involvement in Western patients follow the same pattern described in Eastern populations?

In this issue of *BJS Open*, Blasa *et al*.^3^ retrospectively evaluated 950 patients from five high-volume European centres, representing one of the largest Western multicentre cohorts with detailed station-based lymph node mapping reported to date. Diffuse and mixed histological subtypes demonstrated nearly twice the incidence of nodal metastasis compared with intestinal-type tumours. Subgroup analyses according to tumour location, including fundus, body, and antrum, were also performed. In particular, Figures 4, 5, and 6 provide readers with an interesting visual representation of the risk of lymph node involvement for each nodal station according to histological subtype.

Beyond this already valuable analysis, the authors also explored important contemporary aspects, including microsatellite instability (MSI) status and the impact of neoadjuvant therapy. The lower risk of nodal involvement among MSI tumours has already been discussed in the literature and was once again confirmed in the present study, reinforcing that MSI tumours represent a biologically distinct subset with markedly lower metastatic potential^4^. This finding is especially relevant in the current debate regarding the necessity and extent of D2 lymphadenectomy in patients with MSI, who are frequently older and at higher surgical risk.

The evaluation of patients treated with neoadjuvant therapy is also particularly relevant. Approximately half of the cohort received neoadjuvant treatment, which differs substantially from Eastern series, where most patients undergo upfront surgery, including the historical cohorts that originally defined the risk of nodal dissemination in those populations. The question of how neoadjuvant therapy may influence lymphatic dissemination remains highly relevant. Although this was a subgroup analysis, the present study demonstrated that neoadjuvant treatment appears to reduce nodal tumour burden, an expected and desirable finding, without altering the fundamental pattern of lymphatic dissemination. Finally, the inclusion of nearly 40% of patients undergoing minimally invasive surgery further strengthens the relevance of this study to contemporary real-world practice.

Some limitations of the study should nevertheless be acknowledged. Even in high-volume European reference centres, retrospective assessment of individual lymph node stations carries an inherent risk of inaccurate evaluation. Indeed, detailed nodal mapping was available in only 807 of 950 patients (85%). Accurate dissection of the surgical specimen for precise lymph node station mapping is a crucial, although often underemphasized, step in gastric cancer surgery. Efforts to achieve the same level of rigour and accuracy seen in Eastern pathological assessment of individual nodal stations should remain a priority for Western institutions.

Looking forward, fluorescence-guided surgery may further improve lymph node assessment^5^. In addition to facilitating sentinel lymph node identification, fluorescence imaging may assist intraoperative lymphatic navigation and improve identification of nodal stations, helping ensure adequate lymphadenectomy compliance. Furthermore, this technology may also improve the identification of lymph node stations and individual lymph nodes during postoperative specimen dissection.
